# Brønsted Acid‐Catalysed Dehydrative Substitution Reactions of Alcohols

**DOI:** 10.1002/chem.202002106

**Published:** 2020-10-15

**Authors:** Susana Estopiñá‐Durán, James E. Taylor

**Affiliations:** ^1^ Department of Chemistry University of Bath Claverton Down Bath BA2 7AY UK; ^2^ EaStCHEM School of Chemistry University of St Andrews North Haugh St Andrews KY16 9ST UK

**Keywords:** alcohols, Brønsted acid, dehydrative substitution, homogeneous catalysis, sustainable synthesis

## Abstract

The direct, catalytic dehydrative substitution of alcohols is a challenging, yet highly desirable process in the development of more sustainable approaches to organic chemistry. This review outlines recent advances in Brønsted acid‐catalysed dehydrative substitution reactions for C−C, C−O, C−N and C−S bond formation. The wide range of processes that are now accessible using simple alcohols as the formal electrophile are highlighted, while current limitations and therefore possible future directions for research are also discussed.

## Graphical abstract

**Substitution**: This review outlines the latest advances in Brønsted acid‐catalysed dehydrative substitution reactions using alcohols as the electrophilic component. Progress across a range of different bond formations is covered, while current limitations and possible future directions are also discussed.

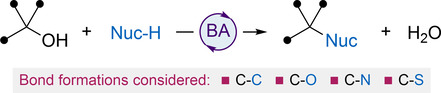

## 1 Introduction

The direct use of alcohols as electrophiles in substitution reactions, releasing water as the only by‐product, is an active area of research both academically and industrially.^[1]^ The availability and tractability of alcohols make them popular substrates throughout organic synthesis.^[2]^ Traditionally, alcohol substrates are stoichiometrically activated prior to substitution, for example by conversion into an alkyl halide or sulfonate ester, overcoming the inherent kinetic and thermodynamic barriers associated with the direct displacement of an hydroxyl group.^[3]^ However, the continued desire to develop new, efficient, more atom‐economical, and sustainable approaches in organic chemistry has resulted in much research into methods for the direct substitution of alcohols. Despite recent advances, the development of new catalytic methods for dehydrative substitution reactions remains an active area of research, as demonstrated by the inclusion of the “direct substitution of alcohols” in the ACS Green Chemistry Institute® Pharmaceutical Roundtable's 2018 update on key green chemistry research areas.^[3]^

**Scheme 1:**
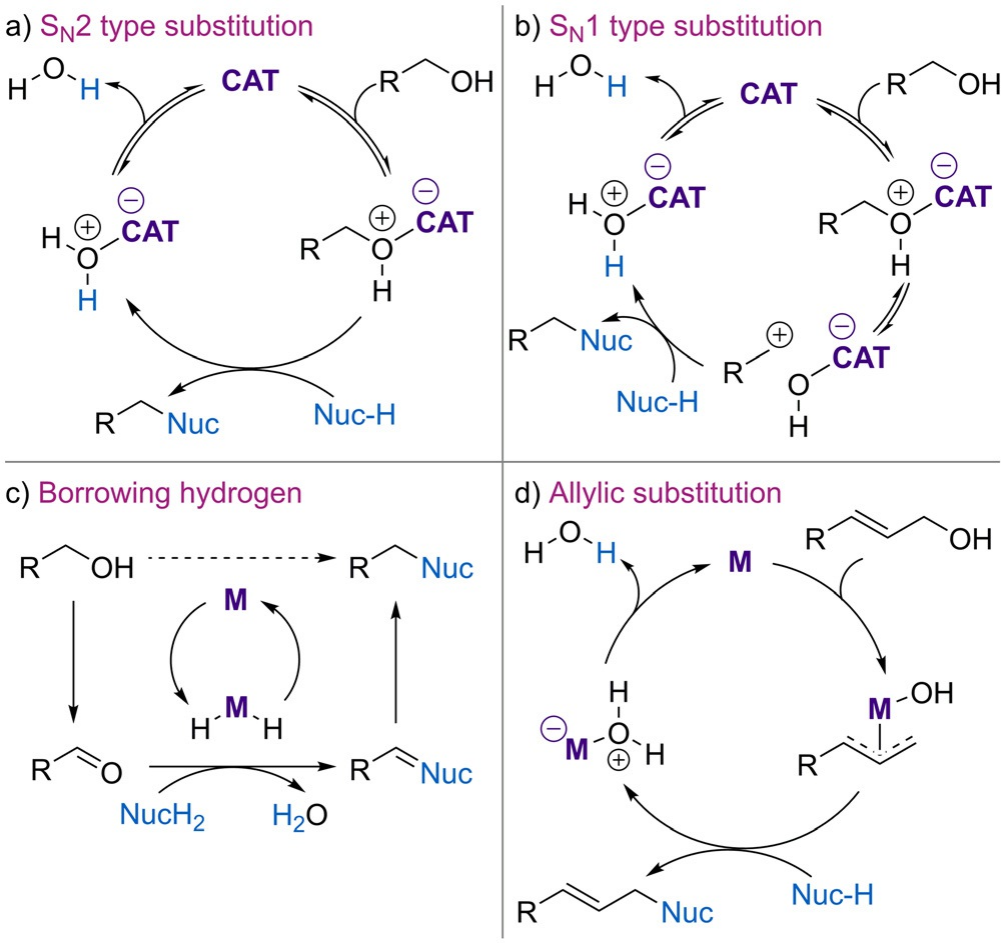
General mechanisms for catalytic dehydrative substitution.

Catalytic dehydrative substitution reactions can occur via traditional S_N_1 or S_N_2 pathways (Scheme 1 a,b), with the catalyst typically interacting with the hydroxyl group to enhance its leaving group ability. Alternatively, metal‐catalysed “borrowing hydrogen” methods allow the formal substitution of primary or secondary alcohols via a redox pathway (Scheme 1 c).^[4]^ There are also a number of metal‐catalysed processes for the direct substitution of allylic alcohols via π‐allyl complexes (Scheme 1 d).^[5]^ For catalytic S_N_1 and S_N_2 processes, various metal‐based and organocatalytic systems have been investigated that operate by many distinct activation modes. For example, a number of metal‐based Lewis acids have been explored, with the comprehensive 2016 review on dehydrative substitution by Moran and co‐workers providing an excellent overview of this area.^[1a]^ The use of Lewis base catalysis for the substitution of alcohols has also been reviewed recently,[^1b^, ^1c^] alongside advances in the development of catalytic Mitsunobu processes.^[6]^

This review highlights recent developments in Brønsted acid‐catalysed dehydrative substitution reactions, with a focus on literature from 2016 up until April 2020. The use of Brønsted acid catalysis often provides alternative and/or complementary reactivity to Lewis acid or Lewis base‐catalysed processes and, to the best of our knowledge, has not been previously reviewed in the context of dehydrative substitution. Brønsted acid‐catalysed dehydrative substitution typically involves protonation of the hydroxyl group to promote an S_N_1‐type reaction, although S_N_2‐type reactivity is also known. Bifunctional Brønsted acid catalysts that also coordinate to the incoming nucleophile are discussed, alongside some dual‐catalytic systems.

## 2 Dehydrative C−C Bond Formation

The formation of carbon‐carbon bonds is fundamental to the synthesis of complex organic molecules. As such, C−C bond formation is the most widely explored dehydrative substitution to date. From Friedel–Crafts alkylation reactions to complex cycloadditions and cascade processes via reactive intermediates, Brønsted acid catalysis has emerged as a powerful method of performing complex dehydrative substitutions for the construction of valuable C−C bonds.

### 2.1 Friedel–Crafts alkylation reactions

Friedel–Crafts alkylation is one of the most important transformations for the functionalisation of arenes and heteroarenes. Significant advances have been made in catalytic Friedel–Crafts alkylations using simple alcohols as electrophiles under both Lewis acid and Brønsted acid catalysis.^[1a]^ Typical Brønsted acid‐catalysed processes are thought to proceed via protonation of the alcohol to promote ionization into a stabilized carbocation, which can undergo electrophilic aromatic substitution. Therefore, this process typically uses electron‐rich benzylic alcohols as the substrate and electron‐rich arenes as the nucleophile. Recent progress in this area has focused on expanding the scope of both reaction partners to include more electron‐deficient substrates.

**Scheme 2:**
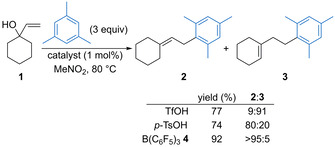
Friedel–Crafts alkylation avoiding isomerisation.

Moran and co‐workers demonstrated that tris(pentafluorophenyl)borane (BCF, **4**) has an advantageous reactivity profile in dehydrative substitution reactions in the presence of acid‐sensitive functionality when compared with other Brønsted or Lewis acid catalysts.^[7]^ For example, Friedel–Crafts alkylation of mesitylene with tertiary allylic alcohol **1** using traditional Brønsted acid catalysts such as triflic acid or *p*‐toluene sulfonic acid generally gave a mixture of **2** and acid‐promoted isomerization product **3** (Scheme 2). In contrast, use of BCF **4** (1 mol %) gave product **2** exclusively in 92 % yield. Mechanistically, the borane may coordinate with water to form a strong Brønsted acid catalyst in situ, but an alternative Lewis acidic pathway could not be ruled out. Meng and co‐workers have subsequently used the same catalyst for the Friedel–Crafts alkylation of phenols with a variety of secondary benzylic alcohols.^[8]^ The same group has extended this to the BCF **4** (10 mol %) catalysed Friedel–Crafts alkylation of electron‐rich anilines with secondary benzylic alcohols.^[9]^ The process is highly solvent dependent, with selective *C*‐alkylation observed in hexafluoroisopropanol (HFIP) and *N*‐alkylation observed in nitromethane.

**Scheme 3:**
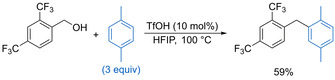
Friedel–Crafts alkylation using electron‐deficient benzylic alcohols.

In 2017, Moran and co‐workers reported the use of triflic acid (10–20 mol %) in HFIP for the Friedel–Crafts alkylation of arenes with electron‐deficient benzylic alcohols, overcoming the previous limitations of such reactions to only electron‐rich alcohol substrates (Scheme 3).^[10]^ The process works well for a range of primary benzylic alcohols, including those bearing highly fluorinated substituents. Notably, the reaction also works with α‐trifluoromethyl benzylic alcohols, forming the corresponding diarylmethane products in generally good yields. Mechanistic experiments suggest that the reaction proceeds via a catalytic S_N_1 dehydrative substitution, with HFIP interacting with the triflic acid to form acidic aggregates capable of promoting ionization of electron‐deficient benzylic alcohols.

**Scheme 4:**
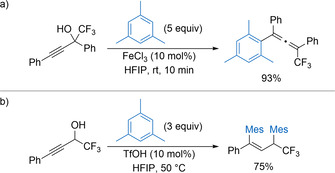
Reactions of α‐trifluoromethyl propargylic alcohols with arenes.

Moran has subsequently published a variety of Brønsted acid‐catalysed reactions of α‐trifluoromethyl propargylic alcohols in HFIP.^[11]^ In the presence of FeCl_3_ (10 mol %), electron‐rich arenes undergo selective γ‐addition to tertiary propargylic alcohols to give tetrasubstituted allenes after short reaction times at room temperature (Scheme 4 a). Extending the reaction time and heating resulted in further reaction of the allenes to form highly substituted indenes after protonation followed by a Nazarov‐type electrocyclization. Mechanistic control experiments suggest that FeCl_3_ in the presence of HFIP forms a Brønsted acid in situ, although the exact nature of the active catalyst remains unknown. Alkenes bearing a *o*‐hydroxyphenyl group undergo an alternative cyclisation, reacting with electron‐rich arenes to form trifluoromethyl substituted chromenes in high yield. In this case, triflic acid (10 mol %) in HFIP was the optimal catalytic system. Secondary α‐trifluoromethyl propargylic alcohols follow a different reaction pathway using triflic acid (10 mol %) as catalyst, giving bis‐arylated alkenes in generally high yields after double Friedel–Crafts alkylation (Scheme 4 b).

**Scheme 5:**
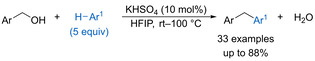
Friedel–Crafts alkylation using potassium bifulfate.

Crousse and co‐workers have also used HFIP as a solvent to enhance the acidity of potassium bisulfate, enabling it to catalyse dehydrative Friedel–Crafts alkylation reactions (Scheme 5).^[12]^ The protocol has been demonstrated for the reaction of a wide range of arenes with various benzylic alcohols, including those bearing electron‐donating or electron‐withdrawing aryl substituents, in generally good yields.

Inorganic phosphomolybdic acid (H_3_PMo_12_O_40_) is an efficient catalyst for the Friedel–Crafts alkylation of 2‐naphthols and benzoheterocycles with substituted benzhydrol derivatives in acetonitrile at room temperature.^[13]^ Hu and co‐workers have also shown that phosphomolybdic acid (3 mol %) catalyses the addition of benzhydrol derivatives across either alkynes or nitriles to form substituted ketones and amides, respectively.^[14]^ Díez‐González and co‐workers have shown that aqueous HBF_4_ is an efficient catalyst for the Friedel–Crafts alkylation of phenols and indoles with secondary propargylic alcohols as the electrophile, while the reaction can also be performed using pentane‐2,4‐dione as the carbon nucleophile.^[15]^

**Scheme 6:**
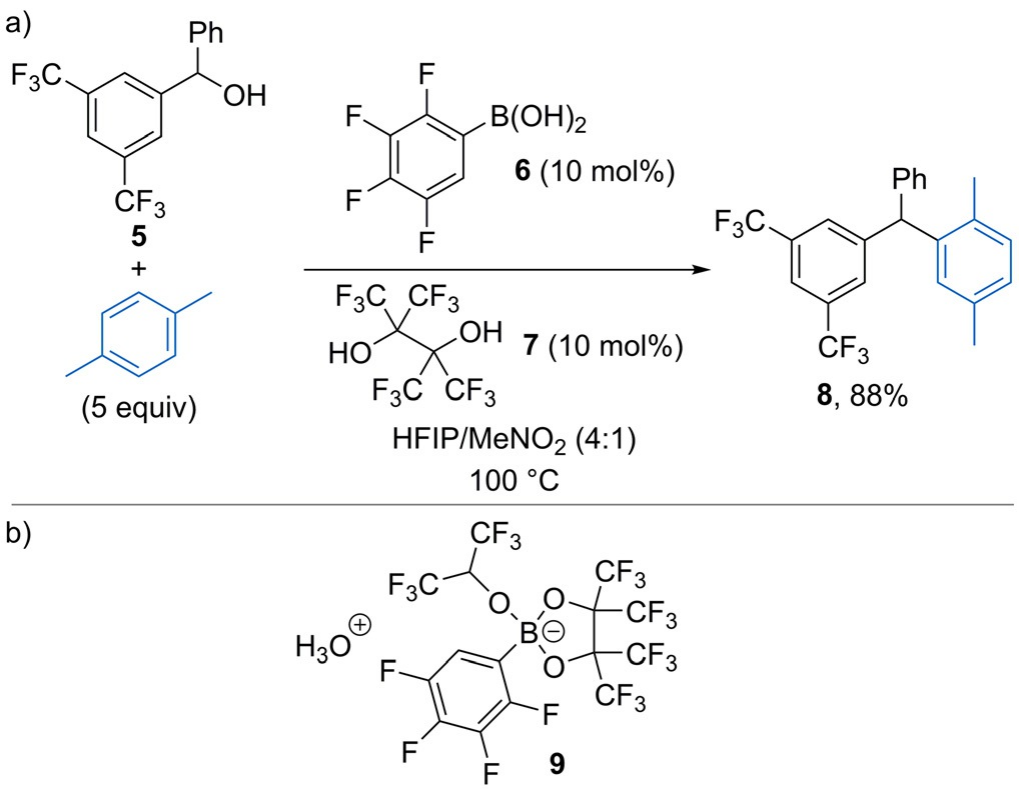
a) Boronic acid‐catalysed Friedel–Crafts alkylation using electron‐deficient alcohols. b) Proposed active catalyst formed in situ.

Arylboronic acids have previously been explored as mild catalysts for dehydrative substitution reactions of alcohols using various nucleophiles.^[16]^ In most cases, these reactions work well with activated allylic alcohols or electron‐rich benzylic alcohols, but do not tolerate substrates bearing strongly electron‐withdrawing substituents. A recent study by Moran suggests that the mode of activation of alcohols using arylboronic acids is likely to be due to either Brønsted acid or H‐bond catalysis, rather than Lewis acid catalysis as initially postulated.^[17]^ Building upon their seminal work in the area,^[18]^ Hall and co‐workers discovered that a combination of 2,3,4,5‐tetrafluorophenylboronic acid **6** (10 mol %) and perfluoropinacol **7** (10 mol %) was effective for Friedel–Crafts alkylation reactions using a range of benzylic alcohols, including those bearing electron‐withdrawing substituents.^[19]^ Perfluoropinacol **7** is essential for the observed reactivity, for example the reaction of *p*‐xylene with alcohol **5** gives product **8** in 88 % yield under the standard reaction conditions (Scheme 6 a), whereas no reaction is observed in the absence of **7**. Mechanistic experiments suggest that boronic acid **6** reacts with **7** and the HFIP solvent to form hydronium boronate complex **9** as the active catalyst (Scheme 6 b), which acts as a Brønsted acid towards the alcohol substrate.

**Scheme 7:**
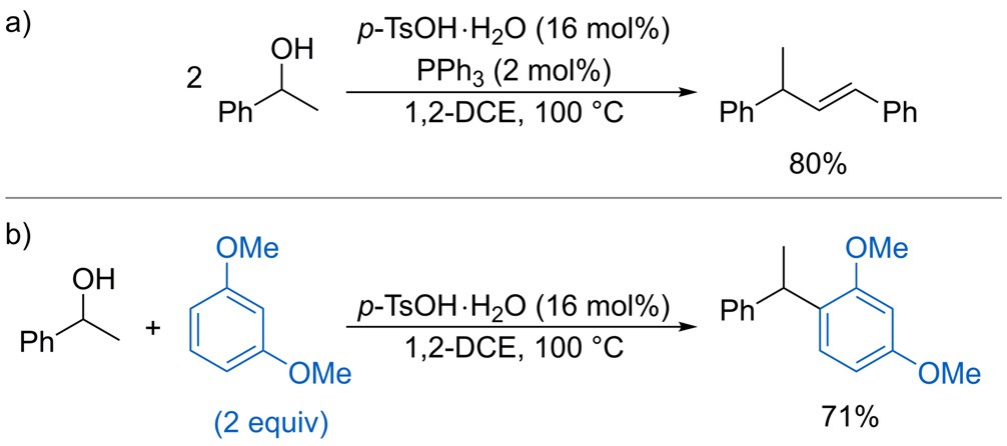
a) Dehydrative homocoupling of benzylic alcohols. b) Friedel–Crafts alkylation using *p*‐TsOH.

Fleischer and Böldl found that electron‐rich secondary benzylic alcohols undergo dehydrative homocoupling in the presence of *p*‐TsOH (16 mol %) and PPh_3_ (2 mol %) to form alkenes (Scheme 7 a).^[20]^ The PPh_3_ is proposed to coordinate to intermediate carbocations to prevent undesired oligomerisation during the reaction. The Friedel–Crafts alkylation of highly electron‐rich arenes is also possible using catalytic *p*‐TsOH (Scheme 7 b).

### 2.2 Generation and functionalisation of quinone methides

An area of dehydrative substitution that has undergone significant developments in recent years is the in situ generation of highly reactive *ortho*‐quinone methides (oQMs) from *o*‐hydroxybenzhydryl alcohols.^[21]^ In particular, the seminal work of the groups of Rueping,^[22]^ Bach,^[23]^ and Schneider^[24]^ showed that chiral phosphoric acids could be used to both generate oQMs and control the enantioselectivity of the subsequent reactions. oQMs formed in this way can undergo a variety of nucleophilic addition and formal cycloaddition reactions.

**Scheme 8:**
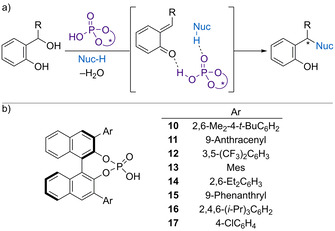
a) Phosphoric acid‐catalysed generation and reaction of oQMs. b) BINOL‐based chiral phosphoric acid catalysts.

In these processes, the chiral Brønsted acid serves two functions; i) to promote dehydration into the prochiral quinone methide and ii) provide a chiral environment for the subsequent nucleophilic addition (Scheme 8 a). Based upon observed product configurations, the chiral phosphoric acids are proposed to act as a bifunctional catalyst, coordinating to both the electrophilic oQM and incoming nucleophile to provide an ordered transition state leading to highly enantioenriched addition products. The use of chiral phosphoric acid catalysts with a BINOL backbone is most common, with a range of different backbone substituents reported (Scheme 8 b).

**Scheme 9:**
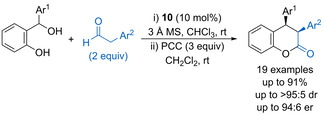
Aryl acetaldehyde addition to in situ generated oQMs.

An early example of a stereoselective addition into an in situ generated oQM used 1,3‐diketones as nucleophiles followed by a cyclodehydration process to form a wide range of 4‐aryl‐4*H*‐chromenes with high enantioselectivity.^[24]^ This concept has since been extended to the use of simple aryl acetaldehydes as nucleophiles to form *syn*‐3,4‐diaryl dihydrocoumarines upon PCC mediated oxidation of the initially formed lactol product (Scheme 9).^[25]^ The protocol uses BINOL‐derived phosphoric acid **10** (10 mol %) as catalyst to form the dihydrocoumarine products in reasonable diastereoselectivity (71:29 to >95:5 dr) and generally good enantioselectivity (83:17 to 94:6 er).

Catalyst **10** is also effective for the enantioselective Friedel–Crafts acylation of indoles and naphthols with in situ generated oQMs.^[26]^ The same general principle can also be used for the enantioselective addition of enamides to oQMs generated from the dehydration of either benzylic^[27]^ or propargylic^[28]^ alcohols to form highly functionalized chroman *N,O*‐acetals bearing three contiguous stereocentres.

**Scheme 10:**
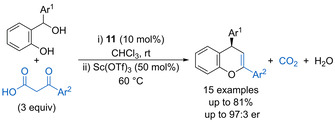
Dehydrative cyclisation of β‐keto acids with oQMs.

Jeong and Kim disclosed that phosphoric acid **11** (10 mol %) catalyses the reaction of β‐keto acids with *o*‐hydroxybenzhydryl alcohols (Scheme 10).^[29]^ Catalytic dehydration forms the corresponding oQM and decarboxylation forms an enol nucleophile, which both remain coordinated to the phosphoric acid to enable enantioselective conjugate addition. Subsequent scandium triflate promoted dehydrative cyclisation forms the substituted 4*H*‐chromene products in good yields with high enantioselectivity.

**Scheme 11:**
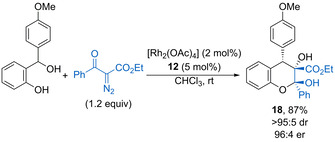
Cooperative phosphoric acid and rhodium catalysed annulation to form substituted chromans.

In 2016, Schneider described a synergistic process combining phosphoric acid and rhodium catalysis for the reaction of α‐diazo‐β‐keto esters with *o*‐hydroxybenzhydryl alcohols (Scheme 11).^[30]^ The transition‐metal catalytic cycle generates a rhodium‐carbene complex, which reacts with the water released from phosphoric acid **12** catalysed oQM formation. The resulting oxonium ylide undergoes an enantioselective conjugate addition to the oQM, controlled by coordination to chiral phosphoric acid **12**. Subsequent hemiacetalisation forms highly substituted chromans such as **18** in good yield as single diastereoisomers with high enantioselectivity. Cooperative chiral phosphoric acid and rhodium catalysis has also been used to form a range of functionalised oxa‐bridged dibenzooxacines in high yields with good stereoselectivity.^[31]^ The reaction proceeds via the [4+3] cycloannulation of in situ generated electron‐rich oQMs and various carbonyl ylides to form complex bicyclic products bearing two quaternary and one tertiary stereocentre.

**Scheme 12:**
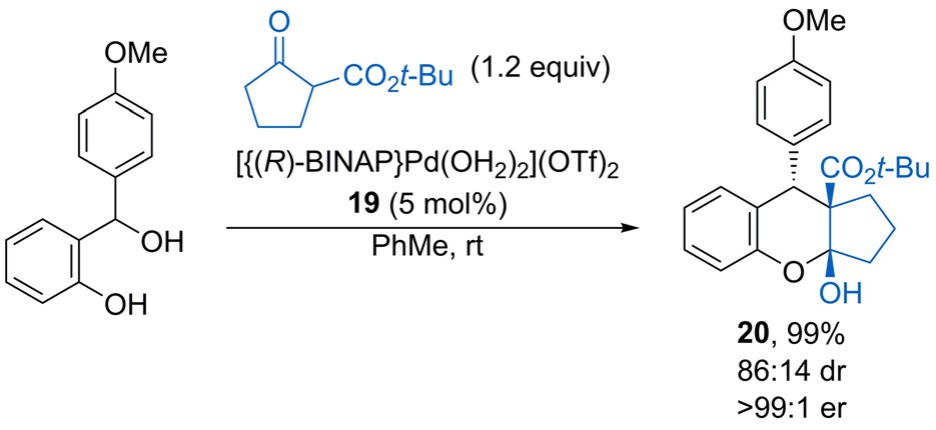
Palladium Brønsted acid‐catalysed annulation of oQMs and β‐keto esters.

Functionalised chromans can also be prepared from *o*‐hydroxybenzhydryl alcohols and β‐keto esters using chiral palladium catalyst **19** (Scheme 12).^[32]^ In this case, the highly acidic aqua ligands help promote dehydration to form the electrophilic oQM, while the cationic Pd complex also forms the nucleophilic chiral β‐keto ester enolate. Conjugate addition followed by hemiacetalisation gives synthetically useful products such as **20** in high yield with reasonable diastereoselectivity and excellent enantioselectivity. Notably, this protocol allows the use of carbo‐ and heterocyclic β‐keto esters, which are not tolerated using phosphoric acid catalysis.

**Scheme 13:**
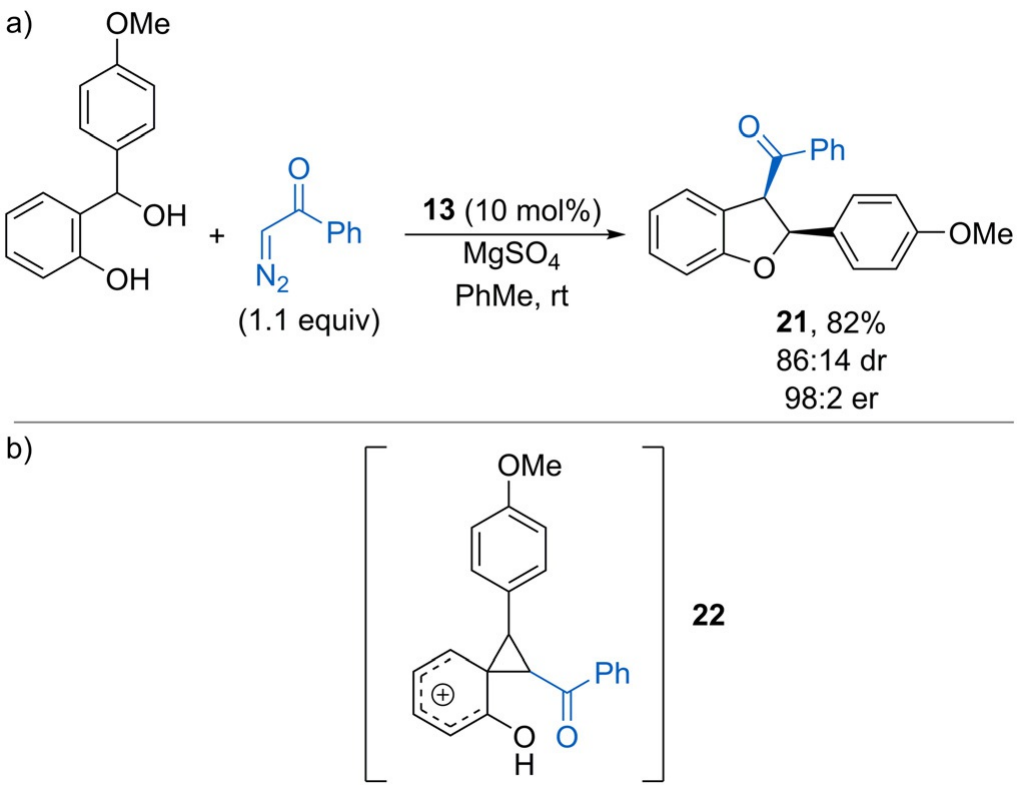
a) Reaction of α‐diazoketones with oQMs. b) Proposed spirocyclic phenonium ion intermediate.

Using phosphoric acid catalyst **13** (10 mol %), electron‐rich *o*‐hydroxybenzhydryl alcohols react with α‐diazoketones to form *cis*‐2,3‐dihydrobenzofurans such as **21** with reasonable diastereoselectivity and excellent enantioselectivity (Scheme 13 a).^[33]^ The unexpected product substitution pattern, with the *C*2 and *C*3 substituents inverted, is accounted for by conjugate addition of the α‐diazoketone into the in situ generated oQM, followed by rearrangement via spirocyclic phenonium ion **22** (Scheme 13 b). Selective ring‐opening of the cyclopropane forms a stabilized benzylic carbocation, which is trapped by the phenol to form the observed benzofuran. The proposed mechanism therefore accounts for the need for an electron‐rich β‐methide substituent.

**Scheme 14:**
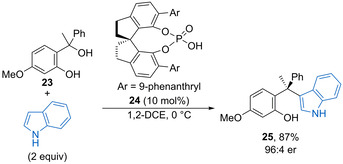
Enantioselective Friedel–Crafts alkylation of indole with oQMs.

Indol can undergo enantioselective Friedel–Crafts alkylation with in situ generated oQMs catalysed by spirocyclic phosphoric acid **24** (Scheme 14).^[34]^ The reaction works for a range of racemic diaryl tertiary alcohol oQM precursors such as **23** with the phenolic ring bearing an electron‐donating substituent. Various benzenoid substituents within the indol ring are also tolerated to form alkylation products such as **25** containing a new quaternary stereocentre in high yield with good enantioselectivity.

**Scheme 15:**
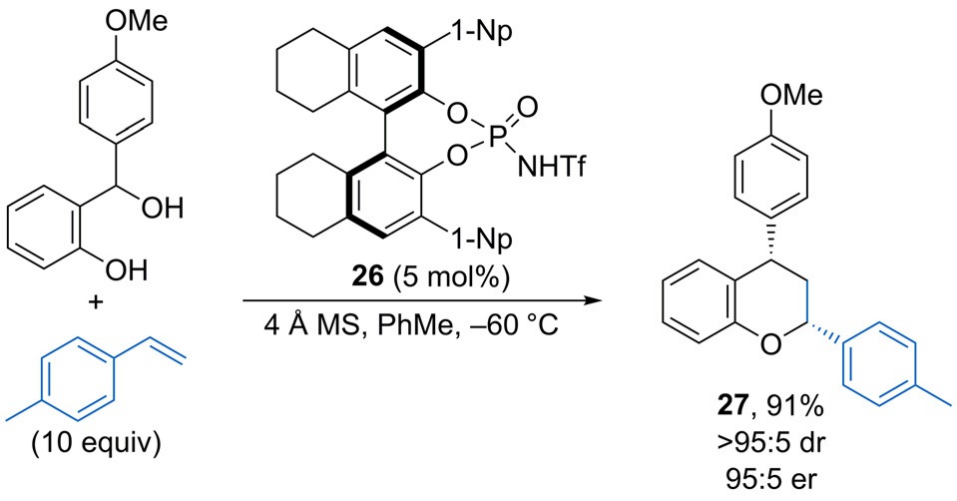
Enantioselective hetero‐Diels‐Alder reaction of styrenes with oQMs.

Processes have also been developed in which oQMs generated through catalytic dehydration participate in cycloadditions.^[35]^ For example, in 2015 Rueping and co‐workers found that *N*‐triflylphosphoramide **26** catalyses enantioselective [4+2] hetero‐Diels–Alder reactions between in situ generated oQMs and unactivated styrene derivatives (Scheme 15).^[36]^ The reaction forms aryl substituted chromanes with generally good yield and high diastereoselectivity, often with excellent enantioselectivity. For example, chromane **27** is formed in 91 % yield as a single diastereoisomer in 95:5 er. The methodology is tolerant of a reasonable range of aryl substituents on either reaction partner and was also demonstrated for the synthesis of 2,3,4‐trisubstituted chromanes. Recently, triflimide has been shown to promote the [4+2] cycloaddition between a wide range of substituted alkynyl thioethers with oQMs to form thioether substituted chromanes in high yields.^[37]^

**Scheme 16:**
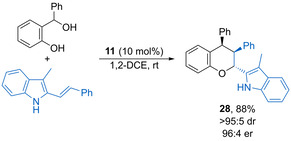
Enantioselective cycloaddition of 2‐vinylindoles with oQMs.

Shi and co‐workers reported that 3‐methyl‐2‐vinylindoles undergo hetero‐Diels–Alder reactions using chiral phosphoric catalysis to form trisubstituted chromans such as **28** with high stereoselectivity (Scheme 16).^[38]^ The 3‐methyl indole substituent is essential for the observed reactivity, avoiding Friedel–Crafts alkylation at this position. The vinylindole alkene geometry was unimportant, with both isomers leading to the same major diastereoisomer with high stereoselectivity, which is proposed to be due to alkene isomerization under the reaction conditions. Readily available 2,5‐dimethylfuran can be used as a dienophile in diastereoselective dearomative [4+2] cycloadditions with oQMs.^[39]^ The substitution pattern of the resulting substituted chromane is dependent on the nature of the catalyst, with (−)‐10‐camphorsulfonic acid and 1,1′‐binaphthyl‐2,2′‐diyl hydrogenphosphate acid giving distinct products.

Zhang and co‐workers showed that an imidodiphosphoric acid catalyst promoted the enantioselective [4+2] cycloaddition between in situ generated oQMs and 1‐((2‐aryl)vinyl)‐naphthalen‐2‐ols to form a range of triaryl substituted chromans in good yield with high stereoselectivity.^[40]^ Independently, Yuan and co‐workers published the same cycloaddition process using a spirocyclic phosphoric acid catalyst, again forming a range of chromans with high levels of stereoselectivity.^[41]^ Schneider and Ukis disclosed the phosphoric acid‐catalysed intramolecular hetero‐Diels‐Alder reaction of in situ generated oQMs with tethered alkenes to form dihydrochromenochromenes.^[42]^ The geometry of the dienophile influenced the stereochemical outcome of the process, with (*E*)‐alkenes giving moderate diastereoselectivity but mostly good enantioselectivity. In contrast, tethered (*Z*)‐alkenes resulted in formation of the fused chroman products as single diastereoisomers but with generally low enantioselectivity.

**Scheme 17:**
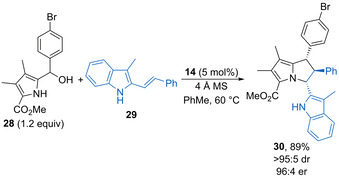
Enantioselective dehydrative [3+2] cycloaddition of 2‐vinylindoles with pyrrole methides.

Schneider and Kallweit have shown that 3‐methyl‐2‐vinylindoles undergo [3+2]‐cycloadditions with in situ generated 2‐methide 2*H*‐pyrroles.^[43]^ For example, chiral phosphoric acid **14** (10 mol %) promotes dehydration of substituted pyrrole **28** to form the corresponding methide, which reacts with **29** to form 2,3‐dihydro‐1*H*‐pyrrolizine **30** in high yield as a single diastereoisomer with excellent enantioselectivity (Scheme 17). The process is tolerant of various aryl substituents to form the complex, highly substituted heterocyclic products with generally high stereoselectivity.

### 2.3 Substitution of indolylmethanols

The use of 2‐indolylmethanol and 3‐indolylmethanol derivatives has also been widely explored in Brønsted acid‐catalysed dehydrative substitution reactions due to their ability to form stabilized carbocation intermediates. These versatile methide‐type intermediates react with a range of carbon‐based nucleophiles to form complex heterocyclic products, in many cases under the control of a chiral Brønsted acid to impart enantioselectivity. The use of indolylmethanol derivatives in catalytic enantioselective synthesis has previously been reviewed,^[44]^ so this section focuses on selected recent developments.

**Scheme 18:**
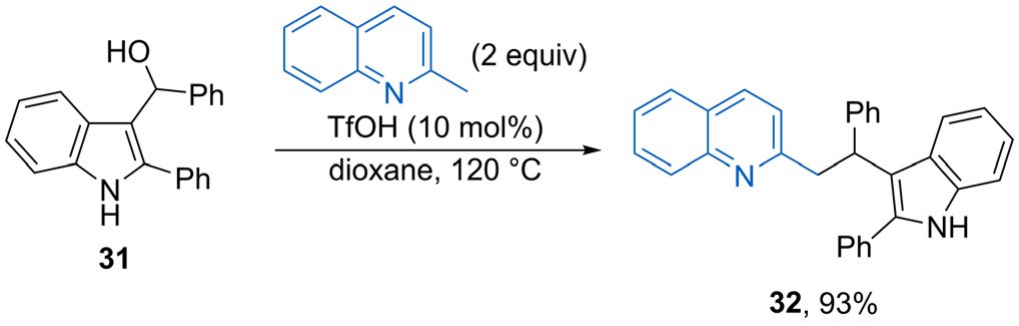
Triflic acid‐catalysed alkylation of 2‐methylquinoline.

Indolylmethanols can undergo acid‐catalysed dehydration to form delocalized vinyliminium ion intermediates, which can undergo regioselective nucleophile attack using different nucleophiles. For example, Xiao and co‐workers have established the triflic acid (10 mol %) catalysed alkylation of 2‐methylquinoline with 3‐indolylmethanol **31** in dioxane at 120 °C to give **32** in 93 % yield (Scheme 18).^[45]^ The reaction works for a range of substituted 2‐methylquinoline and 2‐methylquinoxaline derivatives, although the alcohol component is limited to either substituted 3‐indolylmethanols or activated ferrocenylmethanol derivatives. The acid catalyst is thought to promote both enamine formation from the *N*‐heterocycle and dehydration of the alcohol electrophile to form a stabilized carbocation.

**Scheme 19:**
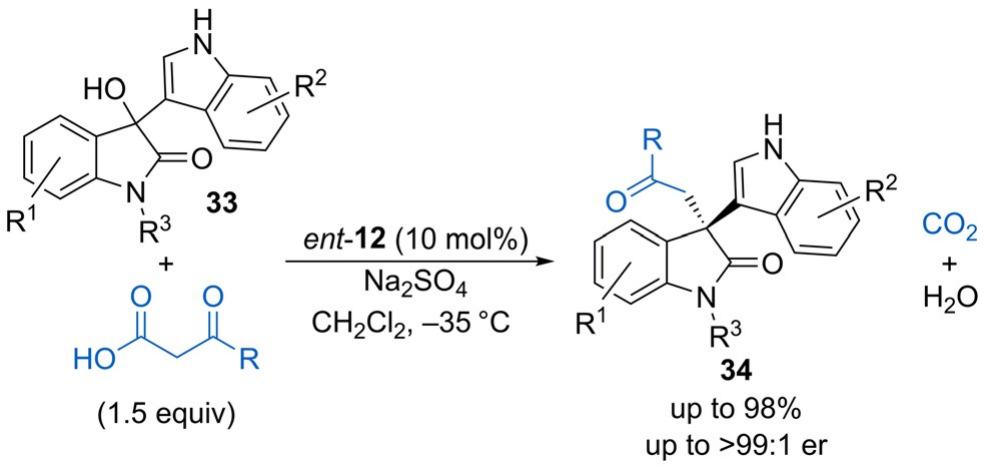
Enantioselective enolate addition to form 3‐indolyloxindole derivatives.

In 2015, Ma and co‐workers described the enantioselective addition of enolates formed from in situ decarboxylation of β‐keto acids to 3‐hydroxy‐3‐indolyloxindoles **33** (Scheme 19).^[46]^ The process is catalysed by phosphoric acid *ent*‐**12**, which is proposed to hydrogen‐bond to the β‐keto acid pro‐nucleophile while blocking one face of the iminium ion generated upon dehydration of **33**. The method allows formation of a range of functionalized 3‐indolyloxindoles derivatives **34** bearing quaternary stereocentres in high yields with excellent enantioselectivity, although the reactions require low temperature (−35 °C) for extended reaction times (36 to 72 h). Xiao and co‐workers have shown that oxindole fused oQM precursors undergo annulation with various 1,3‐dicarbonyls using triflic acid (10 mol %) as the catalyst to form spirocyclic chromen‐oxindole derivatives in good yields.^[47]^

**Scheme 20:**
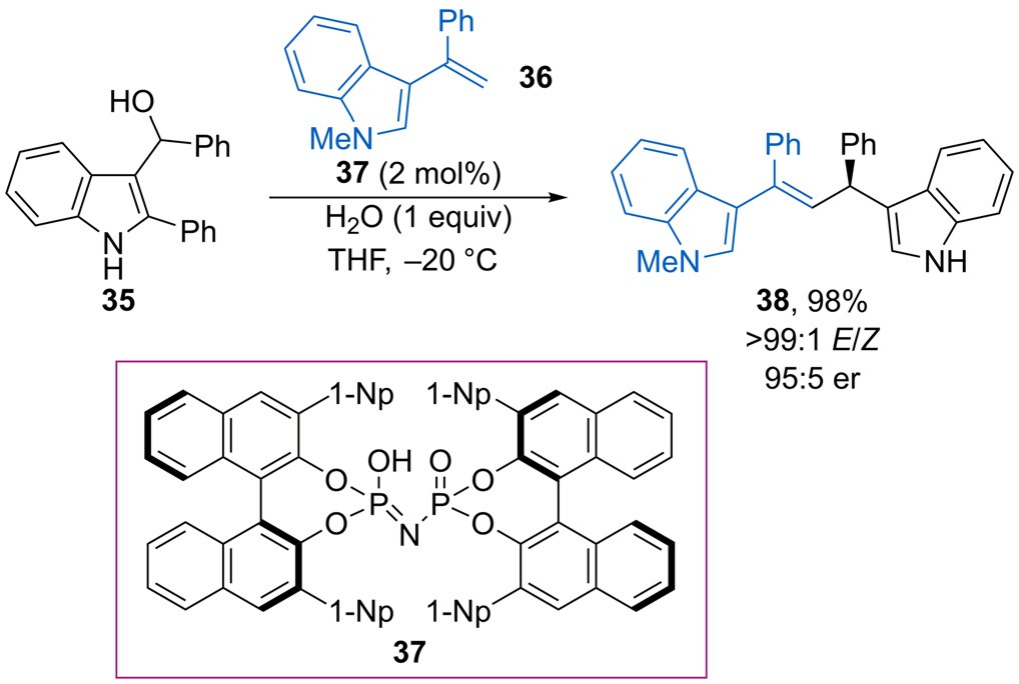
Imidodiphosphoric acid‐catalysed addition of 3‐vinylindoles into 3‐indolylmethanols.

In 2019, Guo and co‐workers found that 3‐vinylindoles are also good nucleophiles for addition into 3‐indolylmethanols.^[48]^ The optimal conditions for the reaction of **35** with **36** use imidodiphosphoric acid **37** (2 mol %) as catalyst, giving product **38** in high yield with excellent stereoselectivity (Scheme 20). The reaction has been successfully tested for a large number of electron‐donating or halogen substituents around each benzenoid ring, while 3‐hydroxy‐3‐indolyloxindoles are also suitable electrophiles. Shi and co‐workers have shown that substituted azlactones can also be used as nucleophiles in diastereoselective dehydrative substitution reactions of 3‐indolylmethanols using readily available diphenyl phosphate (10 mol %) as the catalyst.^[49]^

**Scheme 21:**
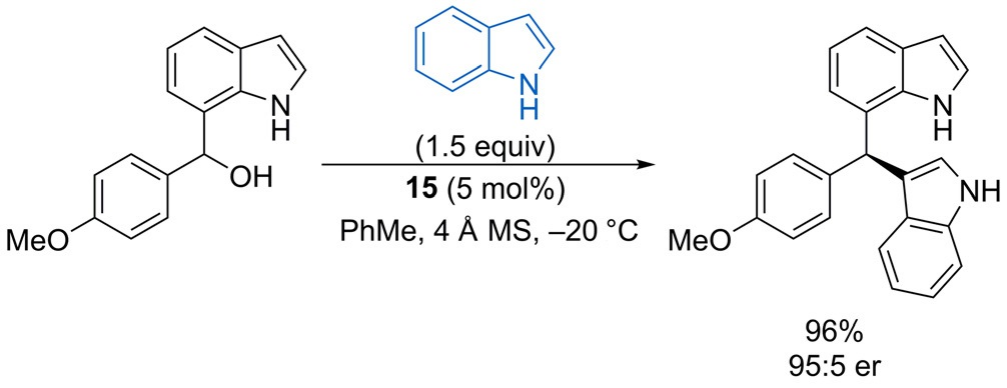
Enantioselective 1,4‐addition of indole to 7‐indolyl methide precursor.

Phosphoric acid catalyst **15** (5 mol %) promotes enantioselective 1,4‐addition of both indole and pyrrole nucleophiles to indolyl methide intermediates generated in situ from 7‐indolylmethanols (Scheme 21).^[50]^ Antilla and co‐workers showed this process works for a range of electron‐rich indolyl methide precursors, forming the triaryl products in generally excellent yield and good enantioselectivity. The method was extended to the use of 6‐indolylmethanols as substrates, providing the products with impressive enantioselectivity for a remote 1,8‐addition. Rao and co‐workers subsequently found that 2‐substituted indoles undergo regioselective 1,8‐addition into vinyliminium intermediates derived from dodecylbenzenesulfonic acid (DBSA) catalysed dehydration of trifluoromethyl 3‐indolyl(2‐thiophenyl)methanols.^[51]^ A wide range of thiophene‐centred bisindole products can be produced in good yields from a range of both reaction partners.

**Scheme 22:**
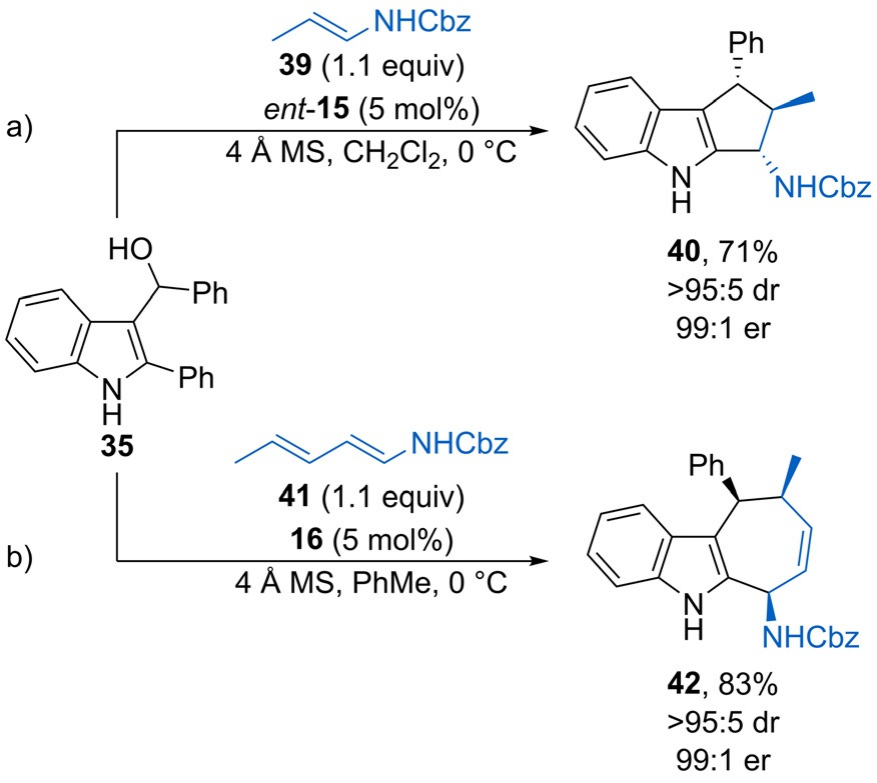
a) Enantioselective formal [3+2] cycloadditions of enecarbamates with 3‐indolylmethanols. b) Formal [4+3] cycloadditions using dienecarbamates.

Masson and co‐workers have disclosed that 3‐indolylmethanols undergo formal [3+2] cycloadditions with enecarbamates such as **39** catalysed by phosphoric acid *ent*‐**15** (5 mol %) to form functionalized indoles **40** with high stereoselectivity (Scheme 22 a).^[52]^ This was subsequently extended to formal [4+3] cycloadditions using dienecarbamates **41** catalysed by phosphoric acid **16** (5 mol %) to form substituted indoles such as **42**, again with excellent stereoselectivity (Scheme 22 b).^[53]^ In both cases, the reaction is tolerant of a range of substituents, forming the polycyclic products in good yields with excellent stereoselectivity. The reactions are proposed to occur via a stepwise mechanism, with dual hydrogen‐bonding activation of the intermediate vinyliminium and enecarbamate required for enantioselectivity.

**Scheme 23:**
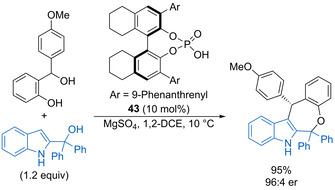
Enantioselective [4+3] annulation of 2‐indolylmethanols with oQMs.

Phosphoric acid catalyst **43** promotes [4+3] annulation of 2‐indolylmethanols with in situ generated oQMs (Scheme 23).^[54]^ The reaction scope is limited to electron‐rich precursors, forming a range of substituted heterocycles in high yields with good enantioselectivity. Mechanistic control experiments alongside DFT calculations support a stepwise reaction pathway, with an initial enantioselective *C*(3) Friedel–Crafts alkylation of 2‐indolylmethanol with the oQM intermediate. Catalytic dehydration of the resulting indolylmethanol generates a carbocation intermediate that undergoes intramolecular cyclisation with the phenol. The reaction enantioselectivity is mostly controlled in the initial Friedel–Crafts addition, with a minor enhancement due to kinetic resolution during the cyclisation step.

**Scheme 24:**
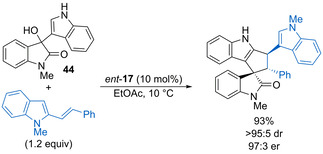
Enantioselective [3+2] cycloaddition of 3‐hydroxy‐3‐indolyloxindoles with 2‐vinylindoles.

A subclass of indolylmethanols that have been explored in various dehydrative substitutions are 3‐hydroxy‐3‐indolyloxindoles, which can lead to the formation of complex polyheterocyclic products. For example, Shi and co‐workers have shown that 3‐hydroxy‐3‐indolyloxindoles such as **44** undergo enantioselective formal [3+2] cycloadditions with *N*‐protected vinylindole derivatives catalysed by phosphoric acid *ent*‐**17** (10 mol %) (Scheme 24).^[55]^ Substitution within the benzenoid fragments of the starting materials with either electron‐donating or halogen substituents is tolerated and, although the majority of the products are obtained in ca. 85:15 dr, the enantioselectivity of the major diastereoisomer is generally high. The presence of an *N*‐protecting group on the vinylindole means that only the alcohol component can be activated by the catalyst, which is in contrast to previous work in which dual activation of both the electrophile and nucleophile is required for high enantioselectivity. The same group has also found that 3‐hydroxy‐3‐indolyloxindoles undergo regioselective cyclisations with Nazarov reagents in the presence of a stoichiometric amount of hydrobromic acid, with either [3+2] or [4+3]‐cycloaddition products formed depending on whether the indol nitrogen atom is protected.^[56]^

**Scheme 25:**
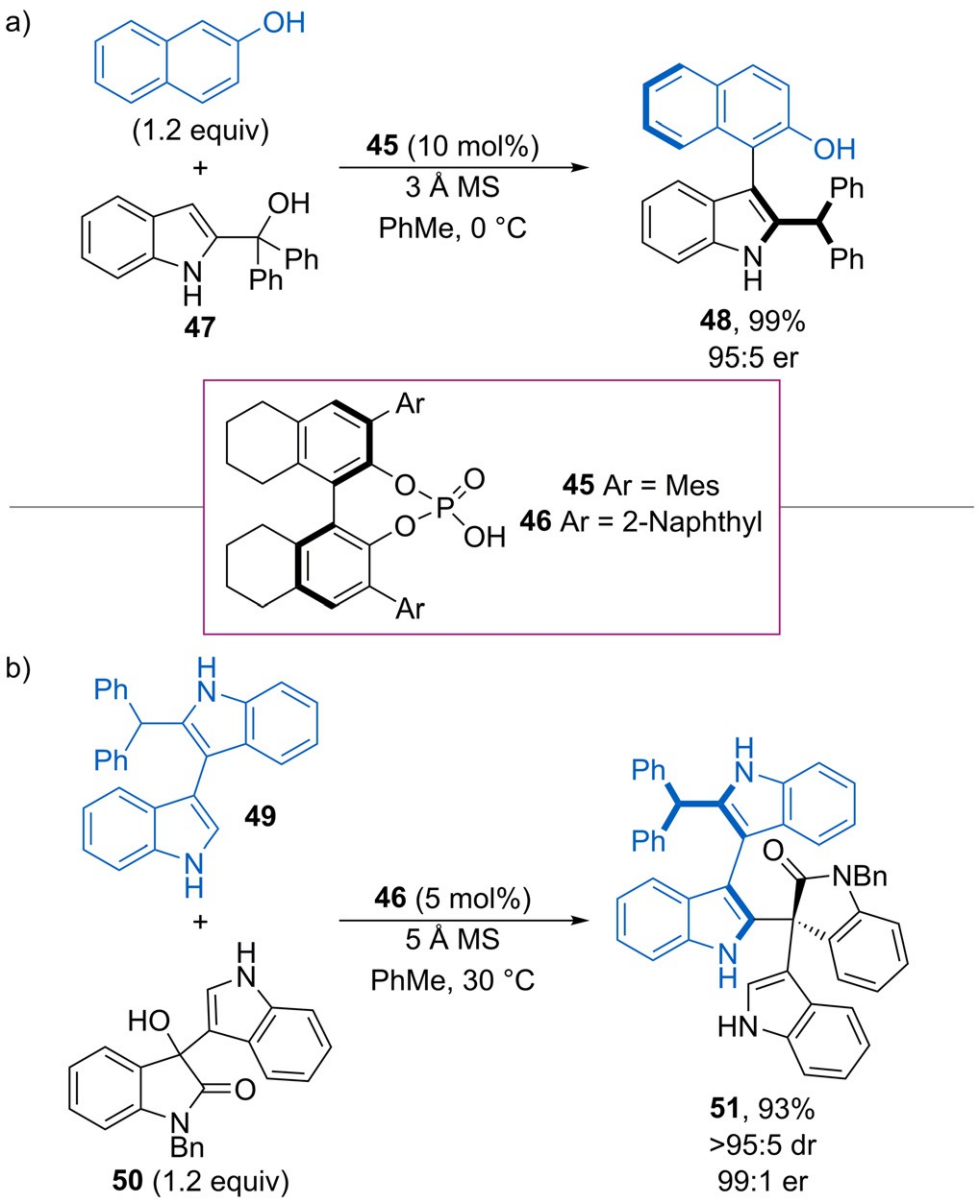
a) Atroposelective addition of 2‐naphthol to vinyl iminium. b) Enantioselective synthesis of substituted 3,3′‐bisindoles.

Shi and co‐workers have reported that phosphoric acid‐catalysed dehydrative substitution of various 2‐indolylmethanols with 2‐naphthols generates axially chiral heterobiaryl products (Scheme 25 a).^[57]^ For example, configurationally stable indole **48** is formed in 95:5 er, with a calculated rotational energy barrier of 32.5 kcal mol^−1^. Mechanistically, dehydration of **47** forms a vinyl iminium intermediate, which undergoes nucleophilic attack at the indole *C*(3) position followed by tautomerization to form **48**. Control experiments show that both the indole NH and phenol OH are essential for high enantioselectivity, suggesting dual hydrogen bonding activation of the substrates with the catalyst. Furthermore, the use of a sterically demanding tertiary alcohol within **47** is essential for the regioselectivity of addition, with the analogous secondary benzylic alcohol undergoing selective reaction at the carbinol centre to form a racemic product. This work has subsequently been extended to the synthesis of highly substituted 3,3′‐bisindole products such as **51** from 3‐hydroxy‐3‐indolyloxindoles **50** (Scheme 25 b),^[58]^ with excellent control of both axial and point chirality for a range of examples.

**Scheme 26:**
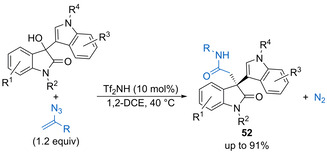
Triflamide‐catalysed addition of vinyl azides to 3‐hydroxy‐3‐indolyloxindoles.

Triflamide (10 mol %) catalyses the addition of vinyl azides to 3‐hydroxy‐3‐indolyloxindole derivatives to form quaternary carbon centres bearing a pendant amide in high yield (Scheme 26).^[59]^ The vinyl azide undergoes dehydrative substitution followed by a 1,2‐migration to release nitrogen and generate a nitrilium ion, which is readily hydrolysed into the amide product. The highly substituted products **52** can undergo reductive cyclisation in the presence of excess LiAlH_4_ to form substituted pyrroloindolinone derivatives in good yield. The process was also demonstrated for a small range of alternative electrophiles, including benzylic, allylic, and propargylic alcohols.

**Scheme 27:**
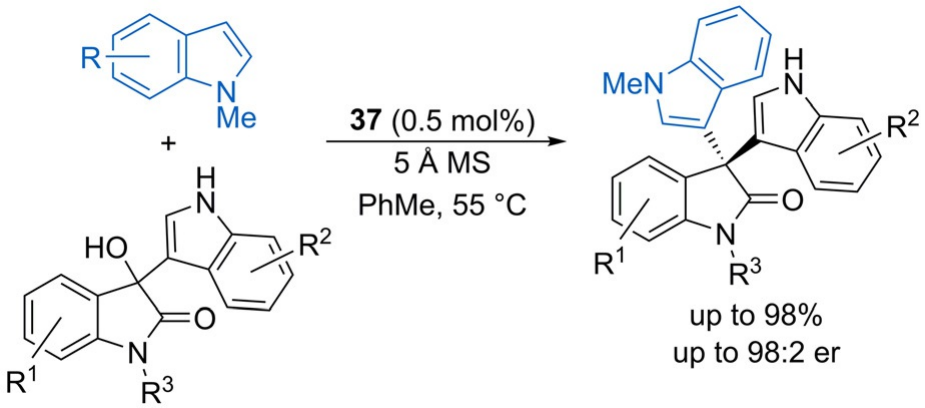
Enantioselective Friedel–Crafts alkylation of indoles with 3‐hydroxy‐3‐indolyloxindoles.

The use of 3‐hydroxy‐3‐indolyloxindoles also allows catalytic asymmetric Friedel–Crafts alkylation of indole nucleophiles to form 3,3′‐bis(indolyl)oxindoles. Shi and co‐workers found that chiral phosphoric acids could catalyse this process to give high yields,^[60]^ although only moderate enantioselectivity was obtained in most cases. Zhang and co‐workers subsequently showed that imidodiphosphoric acid **37** is a highly efficient catalyst for this process,^[61]^ working at low loadings (0.5 mol %), while the addition of 5 Å MS was essential for improving the enantioselectivity without compromising the reaction efficiency (Scheme 27). The reaction scope was demonstrated for substrates bearing a range of substituents within each of the benzenoid rings, while substituted pyrrole nucleophiles are also well tolerated.

## 3 Dehydrative Alkylation of Heteroatoms

The alkylation of heteroatoms is an important process that is widely used throughout both academia and industry. Obtaining selectivity for the desired cross‐alkylation product is one of the key challenges associated with the use of heteroatom nucleophiles in combination with alcohols as the electrophilic component. In this regard, progress has been made across a range of Brønsted acid‐catalysed carbon‐heteroatom bond formations for both inter‐ and intramolecular reactions.

### 3.1 Dehydrative C−O bond formation

**Scheme 28:**
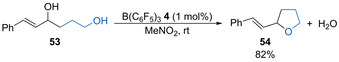
BCF‐catalysed dehydrative intramolecular etherification.

Expanding the reaction scope beyond dehydrative Friedel–Crafts alkylations, Moran and co‐workers showed that BCF **4** could also catalyse inter‐ and intramolecular dehydrative etherification reactions.^[7]^ For example, allylic alcohol **53** bearing a pendant primary alcohol undergoes facile cyclisation at room temperature in nitromethane promoted by only 1 mol % BCF **4** to give substituted THF **54** in 82 % yield (Scheme 28). Meng and co‐workers subsequently expanded the scope of BCF **4** (5 mol %) catalysed intermolecular etherification to the substitution of various secondary benzylic alcohols with both isopropanol and benzyl alcohol used as the nucleophilic component.^[8]^

**Scheme 29:**
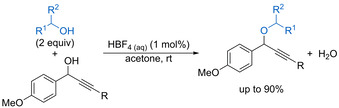
Catalytic intermolecular etherification of propargylic alcohols.

Díez‐González found that aqueous HBF_4_ catalyses dehydrative etherification of propargylic alcohols with a range of primary or secondary alcohols acting as the nucleophile (Scheme 29).^[15]^ The reaction works efficiently at room temperatures for substrates bearing an electron‐rich aryl ring, while higher catalyst loadings and/or higher temperatures are required for neutral or electron‐deficient aryl substituents.

**Scheme 30:**
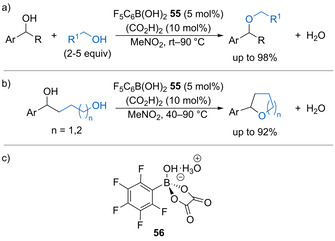
a) Arylboronic acid‐catalysed dehydrative intermolecular etherification. b) Catalytic intramolecular etherification. c) Proposed active Brønsted acid catalyst formed in situ.

In 2019, Taylor and co‐workers published the intermolecular dehydrative substitution of benzylic alcohols to form both symmetrical and non‐symmetrical ethers catalysed by a combination of pentafluorophenylboronic acid **55** (5 mol %) and oxalic acid (10 mol %) in nitromethane (Scheme 30 a).^[62]^ The reaction was tolerant of a range of substituted secondary benzylic alcohols, while non‐participating functional groups including alkenes, alkynes, and esters were tolerated within the nucleophilic alcohol. The same conditions could also be applied to the intramolecular dehydrative cyclisation of diols to form aryl substituted tetrahydrofurans (THFs) and tetrahydropyrans (THPs) in good yields (Scheme 30 b). Control experiments showed that hydronium boronate complex **56** (Scheme 30 c), which was isolated and characterized by X‐ray crystallography, is formed in situ and is likely to act as a Brønsted acid catalyst to promote an S_N_1 substitution process.

**Scheme 31:**
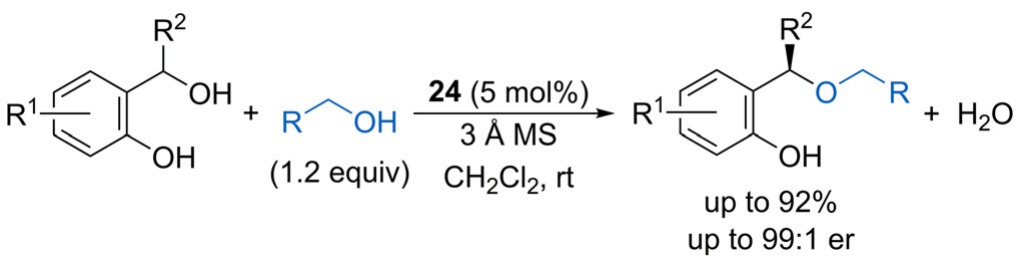
Phosphoric acid‐catalysed enantioselective intermolecular etherification using oQMs.

Building upon the seminal work of Rueping on phosphoramide‐catalysed enantioselective intramolecular dehydrative allylic substitutions to form chromenes,^[22a]^ Sun established that primary alcohols can undergo enantioselective intermolecular addition to in situ generated oQMs.^[63]^ Spirocyclic phosphoric acid **24** (5 mol %) proved optimal, promoting the addition of a range of primary alcohols to substituted oQM precursors in good yield with mostly high enantioselectivity (Scheme 31). The process was tolerant of a range of non‐participating functional groups within the nucleophilic alcohol component including alkenes, alkynes, protected alcohols, and phthalimides, allowing for further derivatization of the products.

**Scheme 32:**
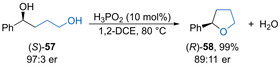
Phosphinic acid‐catalysed intramolecular etherification with chirality transfer.

Samec and co‐workers have described the intramolecular dehydrative cyclisation of pendant primary alcohols onto secondary benzylic alcohols with chirality transfer.^[64]^ A range of Brønsted and Lewis acid catalysts were screened in the reaction of enantiomerically enriched diol **57** to form substituted THF **58**, with phosphinic acid (10 mol %) giving complete conversion to the product with 91 % chirality transfer (Scheme 32). DFT calculations suggest that phosphinic acid acts as a bifunctional catalyst, protonating the secondary alcohol while deprotonating the primary alcohol, to promote a direct intramolecular dehydrative substitution and accounting for the observed inversion of configuration. This is a notable exception to other dehydrative substitution processes that most commonly proceed via an S_N_1‐type mechanism with complete loss of stereochemical information.

**Scheme 33:**
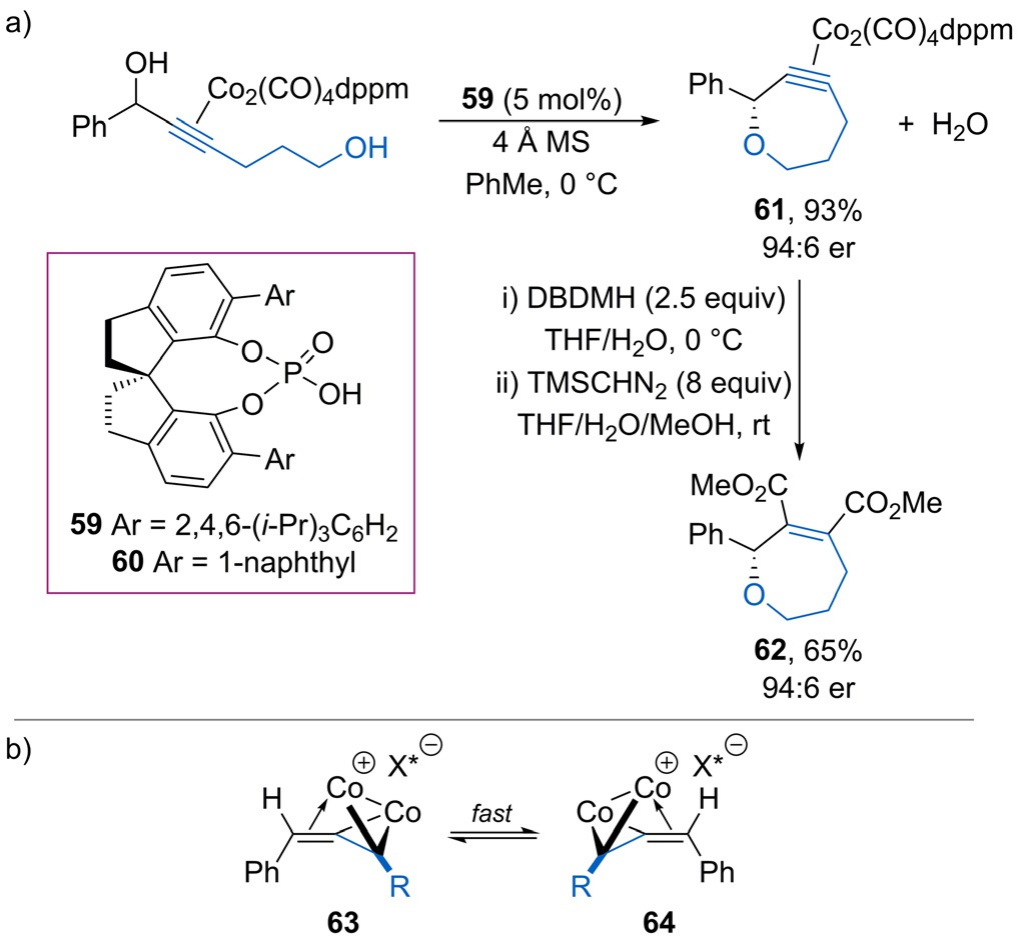
a) Enantioconvergent intramolecular Nicholas reaction of propargylic alcohols. b) Interconversion of intermediate planar‐chiral dicobalt complexes.

Terada and co‐workers have developed an enantioconvergent intramolecular Nicholas reaction of racemic cobalt‐complexed propargylic alcohols with a tethered primary alcohol to form seven‐membered cyclic ethers.^[65]^ Spirocyclic phosphoric acid catalysts **59** or **60** (5 mol %) proved optimal, giving cobalt complexed ethers such as **61** in good yield with high enantioselectivity (Scheme 33 a). The reaction was tolerant of a range of electron‐rich aryl and heteroaryl substituents, giving the products with high yields and mostly high enantioselectivity. Ether **61** could be derivatized by reaction with 1,3‐dibromo‐5,5‐dimethylhydantoin (DBDMH) followed by methylation of the intermediate anhydride to form diester **62** without loss of stereochemistry. The reaction is thought to proceed via planar‐chiral cationic dicobalt complexes **63** and **64**, with the diastereomeric ion‐pairs undergoing rapid interconversion (Scheme 33 b). This allows for an enantioconvergent intramolecular nucleophilic addition to occur controlled by the chiral phosphate counterion.

### 3.2 Dehydrative C−N bond formation

A number of the systems reported for dehydrative C−O bond formation have also been tested with nitrogen‐based nucleophiles. For example, Moran's BCF **4** catalysed dehydrative substitution was successfully demonstrated for the reaction of both *tert*‐butyl and benzyl carbamate to form *N*‐Boc and *N*‐Cbz protected allylic and propargylic amines, respectively.^[7]^ An intramolecular dehydrative amination was also possible to form *N*‐tosyl pyrrolidine in quantitative yield. Díez‐González and co‐workers showed that aqueous HBF_4_ could also catalyse the dehydrative alkylation of carbamates, sulfonamides, and electron‐deficient anilines with secondary propargylic alcohols as the electrophile.^[15]^ Samec and co‐workers intramolecular substitution of secondary alcohols using phosphinic acid (10 mol %) was applicable to tethered aniline nucleophiles, providing the corresponding substituted pyrrolidines in high yield with excellent chirality transfer.^[64]^

**Scheme 34:**
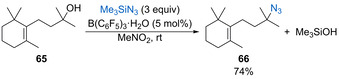
BCF**⋅**H_2_O catalysed azidation of tertiary alcohols.

In 2015, Moran and co‐workers published the azidation of tertiary alcohols using B(C_6_F_5_)_3_⋅H_2_O (5 mol %) as a strong Brønsted acid catalyst.^[66]^ The reaction works for an impressive range of tertiary alcohols bearing a variety of non‐participating functional groups, forming the azide products in mostly high yields. For example, alcohol **65** reacts with trimethylsilyl azide to give functionalized azide **66** in 74 % yield (Scheme 34). The process is also selective for the azidation of tertiary alcohols in the presence of tethered primary or secondary alcohols, with no competing intramolecular cyclisation of the diol starting materials observed. The optimal reaction conditions require the presence of a nitro‐group in the form of either nitromethane as the solvent or 4‐nitroanisole (50 mol %) as a substoichiometric additive in benzene. Kinetic studies show that various nitro‐additives result in a dramatic rate increase compared with the reaction in their absence. Further initial rate studies coupled with IR and NMR evidence suggests that the nitro‐additive forms aggregates with the Brønsted acid in solution, with the active catalytic species likely to contain two molecules of each. The formation of a catalytic supramolecular assembly with various nitro‐additives is also supported by recent DFT calculations.^[67]^ This is an interesting observation that may have wider implications for the mechanism of other Brønsted acid‐catalysed dehydrative substitution reactions performed in nitromethane solvent. Bolshan and co‐workers later described the azidation of both benzhydrol derivatives and protected carbohydrates with trimethylsilyl azide catalysed by HBF_4_⋅OEt_2_.^[68]^

In 2019, Chan and co‐workers showed that electron‐rich primary anilines undergo selective *N*‐alkylation in nitromethane with a range of secondary and tertiary benzylic alcohols catalysed by BCF **4** (10 mol %).^[9]^ Subsequently, Maji disclosed the BCF **4** (1 mol %) promoted dehydrative alkylation of secondary anilines with a range of electron‐rich primary, secondary or tertiary benzylic alcohols.^[69]^ The method was also applicable to the monoalkylation of primary sulfonamides.

**Scheme 35:**
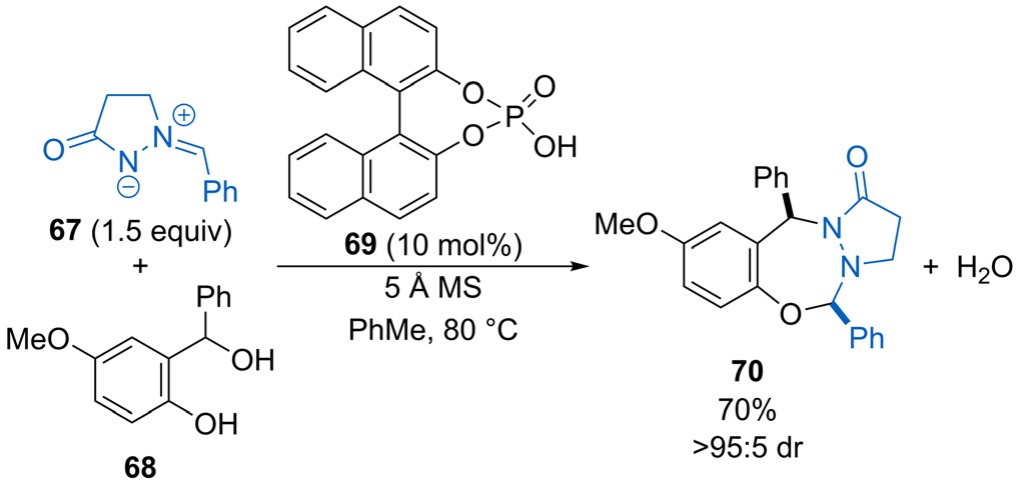
Diastereoselective [4+3] cycloaddition of oQMs with cyclic azomethine imines.

Shi and co‐workers found that in situ generated oQMs from phosphoric acid‐catalysed dehydration undergo diastereoselective [4+3] cycloaddition with cyclic azomethine imines as 1,3‐dipoles.^[70]^ For example, oQM precursor **68** reacts with **67** using 10 mol % **69** to form *cis*‐heterocycle **70** in good yield and >95:5 dr (Scheme 35). The reaction works best for phenolic oQM precursors bearing an electron‐donating methoxy substituent and is tolerant of aryl or alkyl substituents in the benzylic position and aryl substituents within the azomethine imine component.

**Scheme 36:**
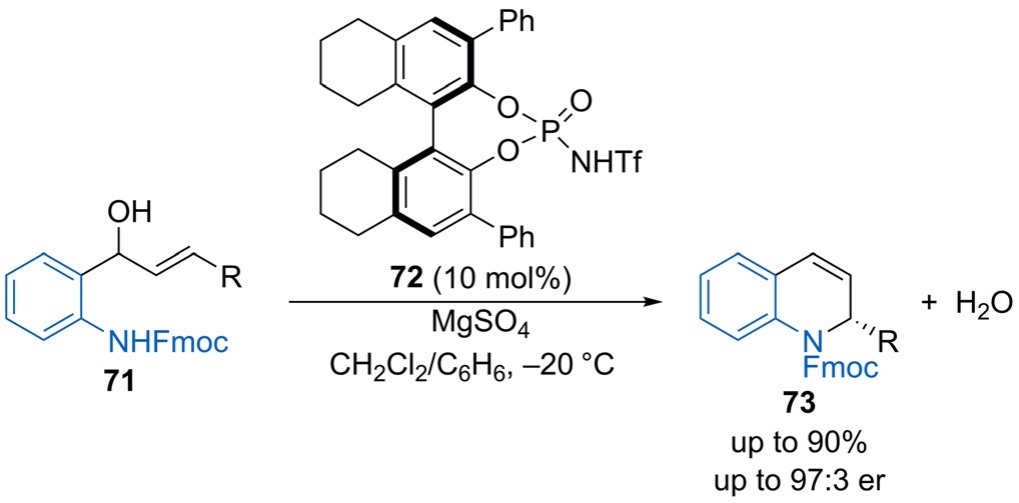
Enantioselective intramolecular amination of allylic alcohols.

Zhou and Xie have shown that chiral phosphoramide **72** (10 mol %) catalyses the intramolecular amination of allylic alcohols **71** to form 2‐substituted dihydroquinolines **73** (Scheme 36).^[71]^ The demonstrated reaction scope includes a range of aryl allylic substituents, forming the products in good yield with moderate to good enantioselectivity.

### 3.3 Dehydrative C−S bond formation

Brønsted acid‐catalysed dehydrative substitution using sulfur‐based nucleophiles has been the least explored of the heteroatom alkylation processes. The BCF **4** promoted substitution of primary, secondary and tertiary benzylic alcohols has been demonstrated using either substituted thiophenols or alkyl thiols as nucleophiles.[^7^, ^8^] The phosphinic acid‐catalysed intramolecular substitution of enantiomerically enriched secondary benzylic alcohols also works with tethered thionucleophiles, forming tetrahydrothiophene products with high yields and high enantioselectivity.^[64]^

**Scheme 37:**
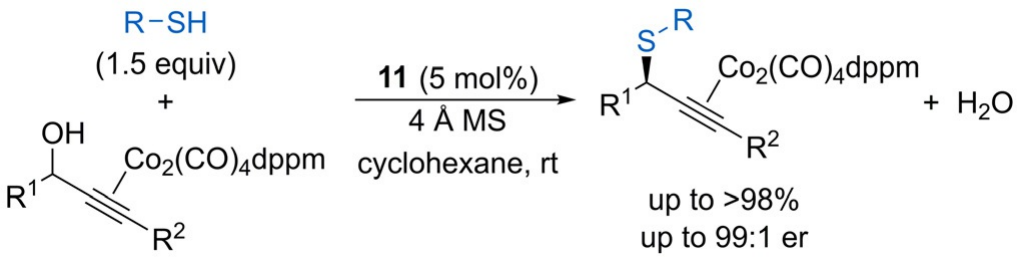
Enantioconvergent intermolecular Nicholas reaction for thioetherification of propargylic alcohols.

Terada and co‐workers have developed an enantioconvergent intermolecular Nicholas reaction for the thioetherification of racemic cobalt‐complexed propargylic alcohols catalysed by chiral phosphoric acids (Scheme 37).^[72]^ The reaction works for a range of substituted propargylic alcohols using either thiophenols or alkyl thiols as the nucleophile to form the corresponding thioethers in excellent yield with generally good enantioselectivity. Mechanistic investigations show that the overall enantioselectivity is dependent upon the reaction concentration and temperature, with the relative rate of racemization of the planar‐chiral cationic dicobalt complexes **63** and **64** (Scheme 33 b) versus nucleophilic addition key to obtaining enantioconvergence. Reducing the reaction concentration helps to minimise premature nucleophilic addition, while higher temperatures (rt versus 0 °C) enhance the rate of racemization and results in higher enantioselectivity.

### 3.4 Dehydrative C−H bond formation

**Scheme 38:**
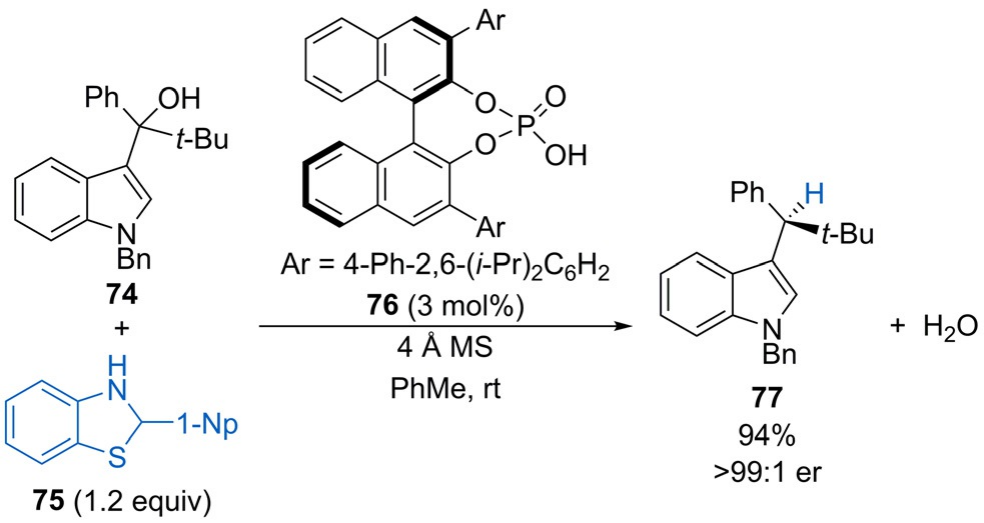
Catalytic reduction of 3‐indolylmethanols to form tertiary carbon stereocentres.

In 2020, Akiyama and co‐workers reported the first example of a phosphoric acid‐catalysed dehydrative C−H bond formation to form a tertiary carbon stereocentre (Scheme 38).^[73]^ For example, phosphoric acid **76** promotes the dehydration of 3‐indolylmethanol **74** and benzothiazoline **75** acts as a hydride source to form product **77** in high yield with excellent enantioselectivity. The reaction works for a range of aryl and alkynyl indolylmethanol substituents although the sterically demanding *tert*‐butyl group is important for obtaining high enantioselectivity.

## 4 Summary and Outlook

Significant progress has been made in the development of Brønsted acid‐catalysed dehydrative substitution reactions for a wide range of bond formations. Over the past five years, improvements have been made for valuable synthetic transformations in terms of overall reactivity and selectivity. There have also been significant breakthroughs in the substrate scope accessible for some processes, for example in the use of electron‐deficient benzylic alcohols in Friedel–Crafts alkylation reactions. A wide range of enantioselective transformations has been developed using chiral Brønsted acid catalysis, in particular for C−C bond formation, while stereospecific S_N_2 type reactions have also been published.

While progress has been made in many areas, there are still a number of significant challenges to be addressed in catalytic dehydrative substitution reactions. For example, many dehydrative substitution processes still require the use of electron‐rich benzylic alcohol substrates as a consequence of the S_N_1‐type reactivity. Broadening the scope of these reactions to electron‐deficient benzylic alcohols is a positive first step towards a more general solution using different classes of alcohol with increasing complexity and sensitive non‐participating functional groups. However, increasing the reactivity of these systems while retaining high levels of selectivity will be challenging. The development of highly enantioselective processes across a range of existing and new reaction classes will remain an active area of research, while continued progress in dehydrative catalytic S_N_2‐type processes with high levels of chirality transfer remains desirable. The use of dual‐catalytic systems and counter‐anion directed catalysis will likely aid in these endeavours.

Increasing the fundamental mechanistic understanding of many of these processes will also be beneficial in making improvements in catalytic turnover, functional group tolerance, selectivity, and industrial applicability. For example, many current dehydrative substitutions require unfavourable solvents such as nitromethane or hexafluoroisopropanol. Increased understanding of the role of these solvents will facilitate the development of more industrially viable protocols. For example, recent work by Moran and co‐workers suggest the formation of supramolecular assemblies between BCF⋅H_2_O and nitro‐additives,^[67]^ while many boronic acid catalysts are likely to form strong Brønsted acids in situ using the solvent.^[17]^ These insights further highlight the importance of the solvent in such processes and will help in the design and study of future catalytic systems.

Brønsted acid catalysis will continue to play an important role in developing new dehydrative substitution reactions. This work will be complimentary to future developments using other catalytic activation modes, allowing the use of simple alcohols as substrates in a wide range of reactions. Ultimately, the aim is to surpass the use of traditional stoichiometric substitution methods and, furthermore, allow the use of alcohol substrates in new unique processes that cannot be accomplished from other electrophiles.

## Conflict of interest

The authors declare no conflict of interest.

## Biographical Information

*Susana Estopiñá‐Durán received an MChem in Chemistry at the University of Valencia (Spain), including a six‐month placement at Loughborough University (England) under the supervision of Prof. Gareth Pritchard studying the synthesis and catalytic activity of bisoxazoborolidone Lewis acids. She then completed her PhD on the “Activation of Alcohols using Boronic Acids” in the group of Dr James Taylor at the University of St Andrews (Scotland). Susana is now working as a Senior Organic Chemist at Infineum UK Ltd in Abingdon (England)*.

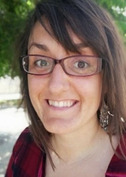

## Biographical Information

*James Taylor received an MChem in Chemistry for Drug Discovery at the University of Bath, before completing his PhD at the same institution under the supervision of Prof. Steven Bull and Prof. Jonathan Williams. He then undertook post‐doctoral research in the group of Prof. Andrew Smith at the University of St Andrews. In 2018, James was appointed as a Lecturer in Drug Discovery at the University of Bath, where his research is focused on the development of sustainable catalytic processes in organic chemistry*.

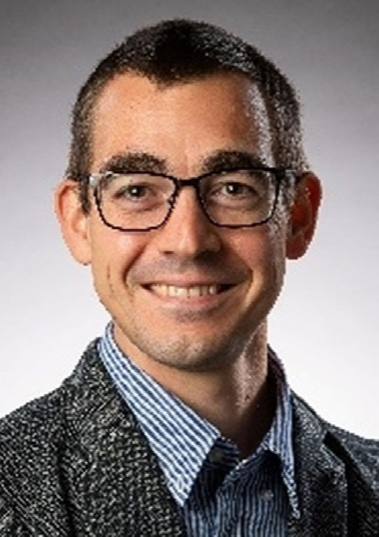

## Supporting information

As a service to our authors and readers, this journal provides supporting information supplied by the authors. Such materials are peer reviewed and may be re‐organized for online delivery, but are not copy‐edited or typeset. Technical support issues arising from supporting information (other than missing files) should be addressed to the authors.

Supplementary
