# Supplementary material for: Brønsted Acid‐Catalysed Dehydrative Substitution Reactions of Alcohols
Source: Chemistry. 2020 Oct 15;27(1):106–20. doi: 10.1002/chem.202002106 (PMC7820959; doi:10.1002/chem.202002106)
Supplement: Supplementary file 1 — Supplementary [file CHEM-27-106-s001.pdf]

Author Contributions

S.E. Writing - Original Draft: Supporting.
